# Tinnitus What and Where: An Ecological Framework

**DOI:** 10.3389/fneur.2014.00271

**Published:** 2014-12-15

**Authors:** Grant D. Searchfield

**Affiliations:** ^1^Section of Audiology, School of Population Health, Centre for Brain Research, Faculty of Medical and Health Sciences, The University of Auckland, Auckland, New Zealand; ^2^Tinnitus Research Initiative, Regensburg, Germany

**Keywords:** tinnitus, model, ecology, adaptation, psychoacoustics, attention

## Abstract

Tinnitus is an interaction of the environment, cognition, and plasticity. The connection between the individual with tinnitus and their world seldom receives attention in neurophysiological research. As well as changes in cell excitability, an individual’s culture and beliefs, and work and social environs may all influence how tinnitus is perceived. In this review, an ecological framework for current neurophysiological evidence is considered. The model defines tinnitus as the perception of an auditory object in the absence of an acoustic event. It is hypothesized that following deafferentation: adaptive feature extraction, schema, and semantic object formation processes lead to tinnitus in a manner predicted by Adaptation Level Theory (1, 2). Evidence from physiological studies is compared to the tenants of the proposed ecological model. The consideration of diverse events within an ecological context may unite seemingly disparate neurophysiological models.

## Introduction

Psychoacousticians and neurophysiologists have, as a rule, approached tinnitus by applying reductionist principles, meaning that the fundamental constituents of tinnitus have been studied in isolation from the overall experience. Traditionally psychoacoustic studies have attempted to control for cognitive effects rather than incorporating them as a requirement for real-world perception (3). Neurophysiological investigations of tinnitus have tended to look at specific loci (4, 5) or the cellular basis (6) of tinnitus generation. Although the complexity of tinnitus has been recognized in neurophysiological models for many years (7) it is now that tools have emerged to enable this intricacy to be examined (8). It has been suggested that, in order to solve tinnitus, principles of systems physiology [a combination of theory, experimental, and computational models (9)] should be applied (10).

Psychoacoustic models of tinnitus have largely been concerned with the elements comprising patient reports of tinnitus sounds (e.g., pitch, loudness) (11–13). There is a need in psychoacoustics to bridge gaps between perception, cognition, and contextual effects (3). The reductionist approaches used in the past may have been necessary for foundation knowledge, but probably underestimate the real-world influences impinging on tinnitus perception. The ecological approach to audition views listening as the experience of auditory events, or objects, not sounds (14, 15). An ecological approach to tinnitus requires that underpinning neurophysiological mechanisms and psychoacoustic outcomes be placed in an environmental context where both the individual’s perception of self and the interplay with the acoustic and social environment are considered. This ecological view challenges us to consider tinnitus as the perception of an auditory object in the absence of an acoustic event. How and where is tinnitus processed as an auditory object when it lacks the features of “true” objects? How does this view take into account the existing fundamental knowledge of tinnitus and physiology? This review attempts to answer these questions by broaching reductionist neurophysiology, and psychoacoustics, with an ecological framework. Ecology in the tinnitus context is defined here as the interactions between an individual and the environment that creates, changes, and continues tinnitus perception.

## Ecological Model

Ecological psychology has many ontologies, it is not the purpose of this review to debate the philosophical basis of this approach to perception (16). The intent is instead to propose a different window through which to view tinnitus. The premise of the model is that we are not passive receivers of sensory information, instead we seek to explain and inform as we move through our environment (17). If we are walking along a road and an ambulance arrives behind us with lights flashing and siren blaring what do we hear? Most people would say “an ambulance” or “a siren” it is unlikely that anyone would say, “two tones 960 and 770 Hz, repeated every 1.3 s” (18). It is normal for us to reference sound to objects or events. In addition, the context of that event will strongly influence our interaction. If the reason we are walking along the street is to search for a child who has not returned home from a bicycle ride the sound of the ambulance will evoke very different reactions than when you are walking to a public open-day by the local emergency services. True sounds have an identifiable physical, object, or source; tinnitus does not. It is a common observation that tinnitus sufferers will seek out the source of their tinnitus sound, only realizing that it is internal through the failure to discover its source in the environment. In tinnitus, the reality of perception is challenged by the absence of an identifiable external source or event (19). Intrinsic factors, environment and interactions will all alter what an individual perceives. The model of tinnitus discussed here consists of three overlapping themes: (1) the individual, (2) environment, and (3) social context.

## Individual

**Figure 1:**
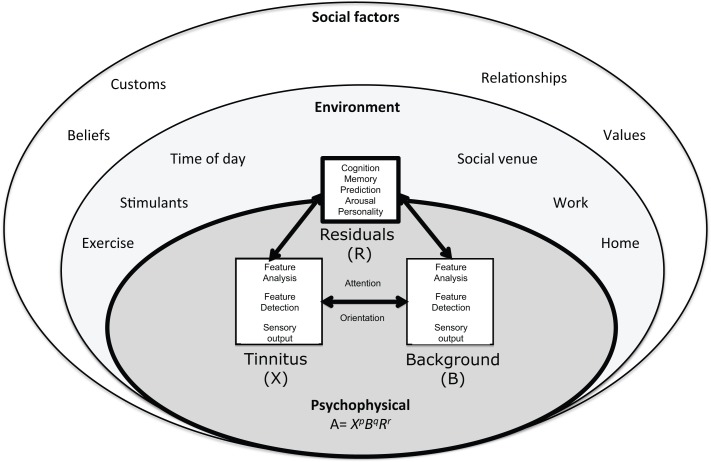
**The ecological model of tinnitus**. This model consists of a psychophysical core described by adaptation level theory in which tinnitus and background sound perception are under influence of individual psychology factors classified in ALT as “residuals.” These factors are influenced by the environment and social context. The adaptation level is the weighted product of: $\bar{X}$, the intensity of tinnitus signal, *B*, intensity of background neural activity, and *R*, intensity of residual components (e.g., memory, arousal, and personality). The weighting coefficients *p, q*, and *r* determine the relative contributions of components to adaptation level and are considered to reflect attention and auditory scene analysis. Helson (1) expressed this relationship mathematically: $A={\bar{X}}^{p}B^{q}R^{r}.$

The individual and their interface with the environment are at the core of this ecological model (Figure 1). Adaptation Level theory (ALT) has been recently hypothesized to explain the relationship between, personality, memory, attention, and tinnitus audibility (2). ALT is a longstanding psychoacoustic theory of perceptual relativity, commonly used to explain and quantify the differences in signal and background in context of experiences (1). Although Helson’s model was developed from laboratory based psychophysics it can be considered ecological in its recognition of the effect of environment and emotion on perception. Tinnitus has been considered in the context of non-auditory modulators before (7, 20); the ALT model of tinnitus differs with an emphasis on neural and psychological adaptation shaping, not only reaction to, but also the perception and magnitude of tinnitus (2).

In this review, adaptation is defined broadly, as “adjustment” (1, 21). An adaptation level or “normal level” exists for all sensory dimensions including perception of frequency and intensity of stimuli (1). The ALT model of tinnitus posits that tinnitus is never static but varies moment to moment with potentially subtle changes in cochlear outflow, emotion, context, and attention. The AL for tinnitus has been previously been proposed as an index of tinnitus audibility (2) but could equally used to explain variations in contributing elements to audibility such as pitch and loudness. Here, an overarching AL of “Magnitude” is considered. If tinnitus magnitude is lower than the AL it is perceived as quiet, higher it is intense. According to the ALT model of tinnitus, the magnitude of tinnitus is determined through the interplay of three external and internal components: (1) the tinnitus signal, (2) background or contextual stimuli, and (3) residuals and social factors (Figure 1). Helson (1) expressed this relationship mathematically:

$$A={\bar{X}}^{p}B^{q}R^{r}.$$

With respect to tinnitus, the formulae may be interpreted in the following manner: *A*, the adaptation level of tinnitus (tinnitus magnitude); $\bar{X}$, represents the intensity of tinnitus signal; *B*, intensity of background neural activity (sensory input); and *R*, intensity of “residual” components (memory, arousal, and personality). Intensity of tinnitus signal and background sensory input is likely to be a function of frequency. The weighting coefficients *p, q*, and *r* determine the relative contributions of components to adaptation level and are considered to reflect attention and Auditory Scene Analysis [ASA (22, 23)]. Although expressed as an equation that, parenthetically, could be solved for tinnitus (2) the equation is used simply here to illustrate the interplay between sound, tinnitus signal, individual psychology, and attention.

## Focus Versus Background

Critical to our understanding of tinnitus is how it is separated from the milieu of other ongoing neural activity, both driven and spontaneous. Hearing must play an important role in this process.

## Hearing

The last 30 years has seen a move away from the view that tinnitus occurs at the cochlea as an increase in spontaneous activity in the eighth cranial nerve (24, 25). Most models now promote changes at the cochlea as a primer for higher order plastic changes (24). Tinnitus onset often begins with hearing loss and difficulties hearing are amongst the most common complaints of tinnitus sufferers (26, 27). Hearing loss and hearing related problems frequently appear as risk factors for more severe tinnitus (28) and the correction of hearing loss through amplification is also an important tinnitus treatment method (29). Both conductive and sensorineural hearing loss are associated with tinnitus, but chronic tinnitus is most often related to sensorineural hearing loss (28, 30). The mechanisms underpinning sensorineural hearing loss are reviewed elsewhere in detail (31, 32). The psychoacoustical consequences of sensorineural hearing loss include: reduced audibility of soft sounds, recruitment, broader frequency tuning, and tinnitus. In some cases, inner hair cell and/or primary afferent damage leaves areas of the cochlear unresponsive to sound [dead regions (33)]. Peripheral hearing damage results in perceptual errors, leading to embarrassment, and social withdrawal (34), as well as central plastic changes such as tonotopic map reorganization (35). Hence hearing loss may both directly affect tinnitus, as an event leading to tinnitus generation, and indirectly through hearing loss related anxiety (36). Hearing loss effects can also be incorrectly projected to tinnitus (37) and become an audible marker for an individuals’ hearing related distress.

The audiogram and changes in auditory threshold are poor indicators of the effect of hearing loss on the individual and when afferent outflow from the cochlea may be altered (38, 39). Tinnitus can occur without measurable hearing loss according to the audiogram (40), but this may be due to the failings of the audiogram to fully detect cochlear and afferent lesions (38, 39). Although the audiogram is only a gross measure of deafferentation, it does have a relationship to the perceived location of tinnitus (41, 42) and tinnitus pitch (43, 44). Tinnitus tends to localize toward the ear with greater hearing loss (42). Tinnitus pitch matches tend to be higher pitched for high frequency hearing loss and lower for hearing loss that extends into lower frequencies (45). Tinnitus likeness matching processes have suggested that tinnitus can be reasonably well replicated as a spectrum that mirrors the audiogram (43). Thus cochlear deafferentation has been hypothesized to lead to perception of sounds as if generated from the deafferented areas. It has been postulated that mechanisms within the ascending pathways attempt to maintain a mean level of activity for homeostasis (46). With a reduction in cochlear-neural activity, gain is applied creating tinnitus as a by-product (47). Gain mechanisms would also be expected to result in a reduced dynamic range to external sounds (47, 48). In humans, loudness growth curves (perceived loudness as a function of intensity) show steeper functions for persons with tinnitus than hearing loss-matched controls (49) consistent with non-linear gain (48). Evidence for neural gain adaptation in individuals with tinnitus is also seen in the Auditory Brainstem Response (ABR). The earliest components of the ABR are reduced in amplitude, but later components increase to normal amplitude, suggesting that loss of sensitivity at the periphery is recovered by later brainstem centers (50). Following noise exposure in animals, there is a decrease in spontaneous activity in the auditory nerve but an increase at the Dorsal Cochlear Nucleus (DCN) associated with tinnitus (51).

While there is evidence that changes in gain accompany tinnitus, it has been argued that other processes are required for tinnitus generation (48). Loudness intolerance may be due to increased gain, and tinnitus a downstream consequence of neural compensation for that gain (48); or loudness intolerance may result from broad hyperexcitability while tinnitus presence is dependent on patterns of remaining spontaneous activity (47). Consequently tinnitus may be dependent on some afferent input (47). The onset of tinnitus and its continuation may draw upon different mechanisms. Chronic tinnitus may not just be a continuation of an acute phase, but may occur through plastic processes following on from the initial pattern creation. Chronic pain, as an example, follows a time dependent process that requires a consolidation phase of hours to weeks (52). The elimination of putative tinnitus generation sites but continuation of tinnitus, such as can occur with cochlear nerve sectioning (53) and removal of the DCN (54), suggests that after ignition tinnitus may consolidate within other centers and networks. Investigating cochlear NMDA-receptor blockade in an acoustic trauma rat tinnitus model Guitton et al. (55) identified a period of 4 days in which intervention was effective, after which it was ineffective, suggesting a shift in the mechanism of tinnitus or possibly consolidation in memory. Robertson et al. (56) observed that hyperactivity at the Inferior Colliculus in a guinea-pig tinnitus model was dependent on cochlear input for up to 8 weeks following cochlear trauma; but after 8 weeks, elimination of cochlear-neural activity with cochlear-cooling, kainic acid, or cobalt no longer reduced hyperactivity suggesting consolidation within the Inferior Colliculus.

A homeostatic response to deafferentation (43, 44) possibly forms the frequency signature of tinnitus and accompanying sensitivity to sound, but additional processes are likely to be required in order to decipher tinnitus as an object.

## Tinnitus as an Auditory Object

Traditionally, we have applied a “musical listening” view to tinnitus, describing tinnitus in terms of frequency and intensity (14, 15) but tinnitus has a complex sound quality that people often describe relative to auditory objects and events (e.g., “crickets,” “cicadas,” and “screaming”) with a spatial location (e.g., left side and in-the-head) (26, 57). Consistent with this observation, several authors have hypothesized that object formation (58) or processes contributing to object formation may play a role in tinnitus perception (59). An auditory object is a thing or event that creates a sound. Auditory objects are important components of ASA (22, 23). In neurophysiology, ASA are those events that encode sound so that they can be separated and recognized from other ongoing auditory activity (22, 23, 58). Two common thoughts experienced by a person upon hearing tinnitus for the first time are “what” is it and “where” is it coming from. Both “what” and “where” sound characteristics are fundamental to ASA, and require the formation of complex semantic and spatial representations in the auditory cortex from simpler spectrotemporal patterns formed in the auditory periphery (23, 60).

Features thought to play a key role in the “what” auditory grouping include: spectral, temporal, and spatial separation, harmonicity, onsets and offsets, coherent amplitude and frequency variations, and bandwidth and phase (61–63). Tinnitus may be coded through many neurophysiological mechanisms that are common with object formation (64). It would seem that parsing out and processing tinnitus from the ensemble activity forming sound would require pattern and target identification by the same cortical and sub-cortical processes employed in ASA (65). Compensatory mechanisms are normally employed by the auditory system to adjust contrast to detect small changes within continuous signals (66). Such compensation may be necessary following the reduction in contrast between external signals and internal noise accompanying hearing loss. To do this auditory neurons in the cortex modify gain moment to moment (67) to code for quiet sounds within an intensity-varying stimulus, and allow response to the sudden appearance of quiet sounds (68). Extracellular recordings in the ferret auditory cortex in response to dynamic random chords (puretones with three different levels of contrast) have shown that the gain of neurons increases for low contrast signals (66). Formation of tinnitus may be achieved by intracortical networks or cortico-thalamic feedback downstream from gain mechanisms in other brainstem centers (66, 69–72).

The localization, or “where,” of auditory events is, arguably, the most important process in ecological psychoacoustics. The perceived location of tinnitus has seldom been considered in the evaluation or treatment of tinnitus, despite the common observation that upon hearing tinnitus for the first time patients often explore their environment to find the source of the sound. The annoying “tinnitus cricket” is discovered not to be an insect only through the individual moving in their environment; as the object does not move closer or further away with head or body movement. Vanneste et al. (73) proposed a tinnitus localization network, on the basis of resting state EEG, consisting of the auditory cortex, angular gyrus, parahippocampal area, and superior premotor cortex. Activity from the premotor cortex appears to differentiate laterality of percept (73). Localization is impaired in persons with tinnitus suggesting either tinnitus results from disruption of neural mechanisms responsible for localization, or the presence of tinnitus makes localization more difficult. In a sound localization test with a seven-speaker array, error scores were higher in persons with tinnitus than controls, and errors in participants with tinnitus on the same side as the speaker were higher than for participants’ with contralateral or bilateral tinnitus (74).

It is reasonable to expect that the perception of tinnitus involves some if not all processes normally applied to identify auditory objects in our environment. But we also know that tinnitus does not follow all rules associated with sound, particularly absence of normal habituation in many sufferers (75) and its unusual magnitude (76). Of particular importance to these tinnitus characteristics may be processes that extract tinnitus as a salient object.

## Tinnitus as a Salient Object

In our daily lives, we are exposed to constant sensory stimulation that requires prioritization and filtering. Intelligible and useful sound information need to be extracted from a background of other competing sounds; unimportant, constant, and familiar stimuli are suppressed once detected (77, 78). Why is it that tinnitus persists when we normally adapt to continuous sensory stimulation? Events that are important to the individual but not clearly understood remain or become salient (79). Stimuli out of context, or unusual, normally engage attention; tinnitus is unusual as a perception because of its “unreality” (19).

An important role of the auditory system in ASA is to integrate knowledge of sensory changes representing the environment with cognitive processes such as memory and attention (22, 23). The saliency and semantics of tinnitus relative to expected auditory activity appears to result in a pop-out effect in which the tinnitus signal is audible, sometimes even when the background sounds are relatively high (80). The auditory thalamus and cortex work together through feed-forward and feedback mechanisms to process ecologically significant aspects of sound influenced by context and arousal (70, 72). The frequencies comprising tinnitus maybe more or less dependent on abnormal gain in response to the deafferented auditory periphery, but tinnitus may only be perceived if a Limbic-Auditory gating mechanism fails to cancel aberrant neural noise (81). Rauscheker et al. (81) suggest that tinnitus perception occurs with a failure of the nucleus accumbens and ventral medial prefrontal cortex to cancel tinnitus generated in lower structures. The role of these structures has preliminary support from fMRI measures of hyperactivity in the nucleus accumbens response to sounds matched to tinnitus (82).

The distinction between signal and masker in normal audition is not solely based on intensity frequency and spatial overlap of sound, but also predictability (83). According to Winkler (83), the auditory system can achieve formation of objects by searching for regulatories (repetitions). This predictive regularity requires encoding, memory, and learning (83). The saliency of auditory events can be determined as a deviation from regularities across dimensions of the sound envelop, harmonicity, spectrum bandwidth, and modulation (84); “salience detection” appears to be a form of bottom-up auditory attention driven by the novel properties of the sound (84). A salient signal related to tinnitus may have been identified in the Ensemble Spontaneous Activity (ESA) from the cochlea. The ESA is the spectrum of “neural noise” recorded at the cochlear round window, or directly from the eighth nerve. The emergence of a peak at 200 Hz in the ESA is seen in tinnitus patients (85) and animals following cochlear lesions that reduce the most predominant spontaneous activity (4). Activity that is repetitive in the auditory periphery, such as the ESA, may be processed as a regularity that conflicts with internal representations of spontaneous activity.

Predictive coding processes have been hypothesized in both the formation of musical hallucinations (86) and tinnitus (87). Errors in the brains interpretation of auditory activity may simply occur as the result of a difference between expected and actual auditory input following hearing loss (88). Kumar et al. (86) hypothesized that the various hierarchical levels of auditory processing try to predict the representation of auditory objects at lower levels; errors in prediction are then fed forward to update representation (86). Phantom auditory perceptions, including tinnitus and auditory hallucinations, can seen to be the result of hierarchical prediction errors or impressions leading to changes in post-synaptic gain (86). Once processed as an object, the absence of an appropriate context may result in a reallocation of attention resources to focus on its perception (87). The aberrant activity may then feed-forward into non-sensory cognitive processes that are strongly associated with tinnitus distress (89). Demand on object processing and attention to tinnitus appears to contribute to a depletion of resources available for other cognitive tasks, potentially explaining impairments in concentration and memory on behavioral tasks amongst persons with tinnitus (90–94). Consideration of the similarities and differences between “hearing voices” and hearing tinnitus may advance our knowledge of both conditions.

## Magnitude

One of the most puzzling aspects of tinnitus has been the apparent paradox between matched tinnitus loudness and severity. Intensity matches of sound to tinnitus usually bare little relation to tinnitus severity or effect on quality of life (76). Although tinnitus loudness appears to share some similar processing with loudness perception, such as the involving the contralateral auditory cortex (95), catastrophic tinnitus can be matched to a low-level external sound (76). Two common explanations for the loudness and severity mismatch have been that: loudness matching methods are erroneous and/or that loudness is independent of severity (96, 97). Although psychoacousticians have attempted to improve pitch match methodology to include or compensate for factors such as recruitment (98) the disparities still exist (99, 100). This may be because loudness ratings and matches to sound are two different constructs and have different reference points. Tinnitus magnitude is the self-perceived audibility of tinnitus, it is not a simple equivalent of intensity but an amalgam of experience and contrast with the context at the time of judgment, and can be considered to be a combination of loudness, severity, and awareness (101). ALT explains magnitude is based on frames of reference or context with top-down influence from psychosocial contributors. In loudness judgments, for example, tinnitus appears greater with less auditory activity (102) and less demand on cognition (103). It is loudness, not intensity, which is normally encoded in the cortex (104, 105). The apparent discrepancy between loudness matching and ratings of “loudness” disappears as a paradox when ALT is applied. Tinnitus matching introduces an external sound as a reference point, while tinnitus loudness ratings are normally undertaken relative to silence, or most recent experienced sound. Aruldasan (106) found a dichotomy of adaptation to perceived and matched loudness after 20 min of silence, broad-band noise at threshold, 10 dB Sensation Level (SL), and 20 dB SL. Measurements were undertaken in silence but immediately following the adapting levels of sound. In the case of the short exposure used (20 min) change in tinnitus loudness ratings were negligible; however, as sound was raised above threshold to 10 and then 20 dB SL the loudness match increased. According to ALT, the matching sound would appear quieter after adaptation to sound and would have to be raised to match the intensity of tinnitus. Aruldasan’s (106) preliminary results would suggest that, at least in the short term, sound adapts to a greater extent than tinnitus loudness. In order for tinnitus loudness matches to sound to be reduced (with a treatment) tinnitus would have to reduce to a greater extent than any sound adaptation. This may explain treatment-related reductions in tinnitus loudness ratings or masking levels but negligible changes in tinnitus loudness matches to sound with time (107, 108). It is not necessarily the psychoacoustical methods that are at fault, but rather their interpretation.

Attention and context of perception also appear to be important factors to manipulate for long-term adaptation to tinnitus. A central component to loudness adaptation has been suggested containing a central feedback loop with gain dependent on peripheral input (109). Tinnitus has been correlated with primary auditory cortex activity; proposed to be due to an “over attention” to audition (110). The magnitude perception component of the ALT model of tinnitus would be consistent with neurophysiological mechanisms in which gain is provided to low-level inputs, and under some top-down control (111). It is possible that a top-down control overlays the gain adaptation to deafferentation, functioning to apply additional gain to salient signals.

## Residuals

In the preceding sections, I have presented arguments as to why tinnitus [*X*, in the (1) ALT model – $A={\bar{X}}^{p}B^{q}R^{r}$] should be considered an auditory object to be extracted from background activity (*B*). It has also been posited that attention and top-down processing (exponents *p, q*, and *r*) contribute to the relative salience of tinnitus to background activity. There are other components to the ALT model; the residuals (*R*). In ALT, residuals are the cognitive, semantic, and psychosocial contributors to magnitude; these include memories of sound, past experience, arousal level, and personality. Residuals may play a very important role in determining tinnitus magnitude.

A role for fear and emotion in tinnitus perception and reaction has been a recurring theme in tinnitus models over the last 25 years (7). Recently, Carpenter-Thompson et al. (112) demonstrated that persons with tinnitus and hearing loss had faster response times to affective sounds than those without tinnitus. The detection and maintenance of tinnitus in the sensory and cognitive domains are believed to interact with individual differences in both motivation and personality to determine response magnitude. There is a considerable body of literature focused on personality in tinnitus (113, 114). As well as playing a role in determining affect, personality may directly influence an individual’s signal detection criterion, and response to sound (106, 113). Welch and Dawes (113) found low social closeness, low self-control, high alienation, and high stress reaction were predictors of tinnitus in a birth cohort assessed at 32 years of age (113). Aruldasan (106) found that the same factors were predictive of sound exposure reducing tinnitus annoyance in a sample of tinnitus sufferers.

Tinnitus is also influenced by individual differences such as health, hearing, coping, and acceptance. Health may influence tinnitus generation directly [e.g., hearing loss, head injury, and ear disease (115)] or its severity through effects on well-being [anxiety and depression; (116, 117)] physical health [e.g., cardiovascular disease (118), arthritis (115), and disability (117)]. Coping and acceptance abilities are also important contributors to how tinnitus affects the individual (119–122). A coping strategy that avoids listening to the tinnitus, and catastrophic thoughts, while seeking social support may result in less disability (120, 121).

Memories and past experience may prime tinnitus affect. Zenner (20) hypothesized that tinnitus induces fear because it cannot be understood in context of an already existing long-term memory; it lacks an established stimulus response pattern. Participants with negative feelings following recall of past frightening experiences report an increase in loudness ratings of tones (123). Similar effects may be seen in tinnitus associated with Post-traumatic Stress Disorder. Sounds may trigger tinnitus and/or stress when those sounds have negative associations (124). While some residual factors are innate to the individual (personality and memories) other residuals, such as arousal will be shaped very much by the individual’s interaction with the environment.

## Environment

Our environment consists of situations (social venues and home) and activities (work and exercise) that result in different states of arousal (stress, joy, and anxiety) and background sounds that vary over the time of day. Day-to-day, minute-to-minute our soundscape changes. With different auditory events come meaning, emotional associations and response. Our environment influences tinnitus audibility, affect, and persistence. Our acoustic world or “soundscape” (125) can influence comfort (126), cognitive performance (127), and potentially health (128); all effects that potentially impact on tinnitus. If tinnitus is seen as being behaviorally important (129) or perceived out of context (19), it will take on greater importance relative to other sounds. The sound levels in different environments (busy office, quiet bedroom, and party) can increase or decrease audibility of tinnitus in a manner predicted by ALT. If there is a reduction in background sound levels or an increased focus on tinnitus there will be a greater weighting to the intensity of tinnitus. Thus the time of day may influence tinnitus magnitude through a time-line of quiet and noisy situations (e.g., waking in quiet bedroom, noisy public transport, quiet office, noisy factory, and quiet home) and accompanying changes in stress levels and emotions.

Certain sounds and activities may impact on emotion and well-being with downstream consequences for tinnitus; attending a concert may induce pleasure and reduce the impact of tinnitus, while relaxation at home may be disturbed by the same music being played loud by the neighbor’s stereo. Tinnitus sufferers may withdraw from social activities for fear of communication problems (37) or concerns that the environments noise or stress may increase their tinnitus. A loss of control over the environment, such as the uncontrollable experience of objects not in the environment (tinnitus), may lead to learned helplessness and poor coping behaviors (130). Such maladaptive coping is unlikely to allow adaptation to tinnitus (121).

The failure to adapt to tinnitus may also result from a disparity in representation across the senses. Sensory input, other than just auditory, may contribute to tinnitus audibility. Our senses usually work together to enable us to interpret our complex environment and confirm the need for action (131). Normally, once an object is formed the need for a response is determined, if no response is needed that signal may be attenuated. Evidence suggests that cross-sensory interactions play a role in human tinnitus (132) and in animal models of tinnitus (133). Multisensory processing in tinnitus perception may be just as important as they are in our exploration of the real world. Hearing can be thought of as an alerting sense, one that can detect objects or events around corners, vision is confirmatory, and tactile perception is interactionary, requiring action within arms reach. Tinnitus alerts us to an object or event, but we cannot confirm its presence with vision and we cannot touch or manipulate it (or for that matter smell or taste it). The presence of an auditory object with no visual or tactile equivalent in the peripersonal space is highly unusual. Burton et al. (134) observed negative correlations in functional connectivity between visual and auditory cortices in persons with tinnitus, possibly because vision is irrelevant to the tinnitus percept. There is some preliminary evidence that sensory-motor incongruence in healthy volunteers can induce pain (135). It is a possibility that auditory–visual incongruence may result, if not in tinnitus generation, in tinnitus disturbance and persistence.

Focusing on the external environment and exploring real auditory objects reduces attention to tinnitus (136). It is a common clinical observation that tinnitus patients will say the tinnitus is less of a problem when they are busy. Involvement in non-tinnitus focused activities such as work or exercise, may change how and if we react to the tinnitus percept. In situations, where attention and higher executive function are directed to non-tinnitus activities (e.g., work) processing of tinnitus may take a lower priority to that when in a low-demand situation (e.g., relaxing at home at the end of the hard day). Cognitive resources are needed to maintain distinction between target and distractor (137); attentional load on non-auditory activities is less likely to see emergence of tinnitus perceptions (103). Conversely, low cognitive load and quiet environment, such as when attempting to sleep, can cause great frustration (27).

## Social Factors

Broader influences than the individual and their immediate environment can be defined as social factors. Tinnitus is an intrinsic experience but it could be strongly influenced by expected behavior and social learning (138). Relationships and support (e.g., family empathy and social isolation) have not been paid a great deal of attention in tinnitus research, but may play a greater role in tinnitus than has been previously considered (139). Some of the response patterns to tinnitus may even be an imitation of another person’s reaction to tinnitus (138). Tinnitus disability is greater when spouses ignore, or become irritated and frustrated with their partners seeking of support (120). The advice that health professionals provide may facilitate coping or prime the system for a negative reaction to tinnitus (120). Societal norms for behavior, culture, and religion may all contribute to tinnitus, as might severe environments such as war and trauma (124, 128). The association between negative thoughts, fear, and tinnitus might be the consequence of classical conditioning (7) or intrinsic learning (20); leading to a sensitization of the CNS and potential lowering of detection threshold (20).

## Connecting the Dots: Ecology, Networks, and Adaptation

Neurophysiology and psychoacoustics provide reciprocal evidence for a complicated multistage change in the auditory system that gives rise to the tinnitus experience. Various homeostatic and adaptation mechanisms appear to accumulate following deafferentation (24). Normal adaptation grows stronger, and becomes more complex along the auditory pathway from the auditory nerve to cortex. Adaptation facilitates a wide range of sounds to be audible or useful while enabling novel sounds to be detected and processed (66). Because adaptation is a constant feature of auditory processing it is likely to play some role in tinnitus induction, consolidation and maintenance. It is the posit of the ecological model described that tinnitus arises through adaptation applied to extract auditory objects from the ensemble of neural activity.

In ecological terms adaptation enables error correction while exploring our environment (1) so tinnitus could be seen as a failure of error correction. The adaptation mechanisms resulting in tinnitus’ unusual annoyance, magnitude, and persistence are likely manifold but could consist of overlapping bottom-up and top-down processes including: homeostatic and contrast gain control (40, 47) bottom-up attention (84) failures against prediction (83, 87) failed noise cancelation (81) and top-down attention modulated gain (87). Deafferentation may result in patterned activity, different from the norm, which can be deciphered as a sound (tinnitus). Gating processes at the thalamus proposed to normally control neural noise, may fail due to influence from the limbic system, allowing further processing (81). If tinnitus is mismatched with predictions in memory, attention should be drawn to the tinnitus (87). This attention is likely to mediate the awareness of tinnitus in that an overly attentive system adapts to extract the tinnitus signal, possibly via a top-down gain mechanism (87, 111). This process may allow extraction of a low-level signal imbedded within neural noise (4).

Semantic processing and an individual’s psychology (response to stress, anxiety, depression, and their personality) will likely contribute to the maintenance or failure to adapt to tinnitus. Object recognition and confirmation would normally require integration of activity from several brain regions, such as those involved in perception, prediction, memory, and emotion. Network models offer a means to relate neural processes subserving the ALT residuals, and the interplay of sensory perception, the individual, and the environment. Imaging studies using differing methodology identify increased connectivity of the auditory cortex, parahippocampus, amygdala, and prefrontal cortex (140–142) consistent with the view that tinnitus is best viewed as a neural network (89). But the imaging evidence is not unequivocal (143).

## Hypotheses and Testing of Model

The primary hypothesis is that tinnitus is the result of complex relationships best examined by considering research within an ecological framework. To test this general hypothesis we need to be cognoscente of how individual neurophysiological components comprising tinnitus combine to form a whole. I suggest two general approaches to research:

1. Longitudinal study of individuals; following individuals in depth over time using a wide range of assessment methods.
2. Build on existing reductionist foundations by adding greater ecological validity, and the study of individual differences within group studies.

The first approach attempts to consider tinnitus as a whole, circumventing the heterogeneous nature of tinnitus, by moving from single measures across groups of sufferers, to multiple measures (objective and behavioral) in single subjects. Such an approach requires a paradigm shift amongst researchers, but also funding agencies and journal editors.

The second approach would attempt to incorporate ecological validity into experiments, while limiting confounding variables. To fully capture the human experience of tinnitus we should use a broad array of assessments, both behavioral and objective. We require more realistic avatars of the tinnitus experience. Psychoacoustic methods should be improved in accuracy and be able to capture individual variability. Recent “tinnitus spectra” methods of tinnitus assessment are a move in this direction (43, 44). Virtual reality offers a potential means to further advance psychoacoustic assessment and render more realistic avatars for tinnitus (144). Virtual environments for fMRI (145) may also assist in capturing more realistic representations of neurophysiological events underpinning tinnitus. Animal models of tinnitus need to, and are, becoming more representative of tinnitus in humans, but could, by way of example, make use of aged animals (146).

The second approach could expand our foundation knowledge through consideration of interactions between ALT components. It is hypothesized that: “Individual differences in personality, emotion, and memories will influence: the perceived magnitude of tinnitus, and the effect of sound and the environment on tinnitus.”

Tinnitus will be able to be modeled using Helson’s mathematical expression of ALT (1) $\left(A={\bar{X}}^{p}B^{q}R^{r}\right)$ by probing individual differences in personality, emotion, and memory while controlling other model components.

Despite criticisms of current methodology existing findings can be integrated within an ecological framework. Many pieces of the tinnitus puzzle seem to be present; an ecological approach may assist in solving the puzzle.

## Summary

It has been proposed here that the unusual magnitude and persistence of tinnitus can be modeled by ALT as adaptive interactions between residual factors, context, and processes extracting “what” and “where” patterns from the environment. The hypothesized neurophysiological basis of the model is that:

- Following peripheral injury central gain increases to compensate for deafferentation (47). The amount of gain is under top-down control.
- Multiple schema-based pattern recognition mechanisms shape tinnitus as an auditory object. Complex networks (89) linked to our individual psychology and semantic processing interacting with the environment apply top-down gain mechanisms such as contrast control to extract salient signals from neural noise (66).
- The tinnitus object is incongruent with predicted patterns (87) due its intrinsic novelty and top-down adaptive attention control tinnitus fails to be canceled (81).
- Interaction with our environment shapes tinnitus processing through variations in background activity, stress, and emotion. ALT explains many of the complex influences of individual psychology and environment on perceptual relativity.

## Conclusion

An ecological model that includes ALT appears to be a useful framework to understand the complex relationships between putative tinnitus mechanisms. Advocacy for an ecological approach to tinnitus should not be misinterpreted as an attempt to discredit or relegate the importance of fundamental neurophysiology; rather it provides a different perspective. Most of the evidence provided in this review to support an ecological approach is indirect and based on reductionist research approaches. The model and its assumptions can, and are, being empirically evaluated. In conclusion, I suggest tinnitus should be defined as the perception of an auditory object in the absence of an acoustic event.

## Conflict of Interest Statement

The author declares that the research was conducted in the absence of any commercial or financial relationships that could be construed as a potential conflict of interest.
